# Application of artificial intelligence and psychosocial functioning in psychosis: a systematic review and meta-analysis

**DOI:** 10.3389/fpsyt.2025.1692177

**Published:** 2025-11-05

**Authors:** Chloe Ho Yee Mok, Calvin Pak Wing Cheng, Menza Hon Wai Chu

**Affiliations:** ^1^ Department of Psychiatry, The University of Hong Kong, Hong Kong, Hong Kong SAR, China; ^2^ Occupational Therapy Department, Kwai Chung Hospital, Hong Kong, Hong Kong SAR, China

**Keywords:** AI, artificial intelligence, machine learning, psychosis, psychosocial functioning

## Abstract

**Introduction:**

Artificial intelligence (AI) has emerged as a valuable tool in mental health care, with applications in the treatment of psychosis. However, its application to psychosocial functioning in psychosis remains underexplored, despite its critical role towards long-term therapeutic outcomes and recovery. The goal of this systematic review and meta-analysis is to identify, summarize, and evaluate the current evidence on AI applications in psychosocial functioning in psychosis.

**Methods:**

A literature search was conducted using the PubMed, Scopus, and ACM Digital Library databases for articles published between January 2010 and March 2025, in accordance with the PRISMA guidelines. Quality of studies was assessed using the Prediction model Risk Of Bias Assessment Tool (PROBAST), Newcastle-Ottawa Scale (NOS), and the Cochrane Risk of Bias Tool (RoB2.0). Meta-analyses synthesized commonly used performance metrics using random-effects models, with subgroup, sensitivity and publication bias analyses.

**Results:**

A total of 14 studies were included in this review. Various AI techniques were employed, with supervised machine learning being the most predominant. Psychosocial domains, including social function, occupational function, social cognition and quality of life, were targeted. Meta-analysis revealed moderate discriminative and predictive accuracies of AI models: pooled AUC of 0.70 (95% CI: 0.63–0.76) and RMSE of 8.15 (95% CI: 7.32–8.98). Subgroup analyses indicated higher predictive accuracy for social cognition (AUC=0.77) and clinical symptom-based predictors (RMSE=7.1), with substantial heterogeneity mainly attributed to methodological variability.

**Conclusions:**

This review discovered the current application of AI in psychosocial functioning in psychosis, including the techniques usage, modeling approaches, targeted domains, and model performance. AI showed promise for early identification, continuous monitoring, and personalized interventions, driven by methodological advances such as ensemble learning with feature selection. Nevertheless, limitations in methodological consistency, data quality, model design, and ethical issues underscore that the field remains in its early stages. Overall, AI should complement clinical expertise, rather than replace it, in delivering psychosocial care in psychosis.

**Systematic review registration:**

https://www.crd.york.ac.uk/prospero/, identifier CRD420251051952.

## 1 Introduction

Psychosis is a mental health condition characterized by a range of symptoms including hallucinations, delusions, and disorganized thinking (1). Apart from the core symptoms, a substantial portion of individuals with psychosis encounter considerable impairments in psychosocial functioning, defined as the capacity to carry out everyday activities and to engage with others and the community in a mutually satisfying way (2–6). The key components comprise social functioning, occupational functioning, quality of life (QoL), and role performance (2, 4). In particular, individuals with psychosis suffer from a decline in cognitive function, including social cognition (7, 8). Social cognition, which encompasses aspects of facial social perception, facial emotion recognition, and theory of mind (ToM), involves the cognitive processes of perceiving, interpreting, and reacting to social information (9, 10). Impaired social cognition can impact one’s performance in routine activities and employment, leading to lower life satisfaction (11–13). However, the subtlety of psychosocial deficits is less apparent than overt psychotic symptoms, complicating early detection and intervention (14). Also, the effectiveness of conventional approaches, such as psychotherapy, social cognition, and interpersonal skills training, was found to be limited by trajectories in psychosis and clinicians’ subjectivity (15). These highlight an urge for innovative, precise, and scalable approaches to manage psychosocial impairments in psychosis.

Over the past decade, Artificial Intelligence (AI) has emerged as a transformative tool in healthcare. Research & Markets (16) projects a remarkable compound annual growth rate (CAGR) of 49.1% for AI in the healthcare market over the next five years, signaling a paradigmatic shift in how medical care will be delivered and offered. AI is defined as a branch of computer science that centers around developing algorithms and systems to replicate human cognitive activities, such as problem-solving, reasoning and learning (17). Common subfields include machine learning (ML), deep learning (DL), natural language processing (NLP), and reinforcement learning (RL) (18). ML, DL, and NLP are capable of analyzing large datasets to determine patterns and characteristics, while RL excels at learning optimal behaviors based on trial and error (19–21).

In the realm of mental health, a growing number of research studies are highlighting the emerging role of AI. For instance, ML has shown potential in forecasting the risk of relapse and violent behavior by analyzing behavioral features (22, 23). NLP was used to evaluate the mental states and functioning of patients by analyzing their patterns in daily life and interpersonal interactions (24–26). Furthermore, AI has contributed to more tailored and interactive interventions, like psychological support and psychoeducation delivered through chatbots (27–29). Given these capabilities, there is a substantial potential for AI to facilitate the diagnosis, monitoring, and treatment of psychosocial functioning in psychosis, offering objective and efficient solutions that traditional methods fall short of.

Although AI has been widely utilized in psychosis research, its application to psychosocial functioning remains largely uncharted. Existing literature primarily focuses on the diagnosis and detection of psychotic disorders. For example, a systematic review examined the current evidence on applying AI techniques for detection and classification in schizophrenia (30). On the other hand, some researchers investigated the use of ML to analyze structured data for diagnosis (31, 32). Additionally, several studies have explored the use of computer vision models to detect biomarkers and forecast progression in brain imaging analysis for the psychosis population (33–35). Whilst some empirical evidence suggested the use of AI during the stage of rehabilitation in the schizophrenia population, most studies concentrated on the prediction of medication adherence and relapse (36). After gathering and reviewing the current literature, a knowledge gap was identified in the application of AI to psychosocial function in psychosis. Given that psychosocial functioning is a fundamental indicator of illness course, therapeutic outcomes and recovery in psychosis, deficits in this domain will not only hinder one’s prognosis, but also impose a substantial burden on healthcare systems (11, 37, 38). Despite the critical need for advancement in managing psychosocial deficits in psychosis, no systematic review has been done to synthesize and consolidate evidence on this aspect. This research gap accentuates a vital opportunity to explore how AI can contribute to patients’ psychosocial recovery.

## 2 Aims of the review

The aim of this systematic review and meta-analysis is to comprehensively identify, summarize and evaluate the current evidence regarding the application of AI to psychosocial functioning in psychosis. following research questions:

- How are AI techniques applied, and which psychosocial domains are targeted?
- What is the performance of these AI techniques?
- What are the potential benefits and limitations of clinical integration?
- What are the key areas for further research?

## 3 Methodology

### 3.1 Search strategy

This systematic review and meta-analysis is registered with PROSPERO (CRD420251051952). A literature search was conducted in three electronic databases: PubMed, Scopus, and ACM Digital Library. The systematic review was in accordance with the PRISMA guidelines. The timeframe of published articles was limited to the last 15 years, between January 2010 and March 2025. The search criteria included the following keywords: (Artificial intelligence OR AI OR Machine learning OR Natural language processing OR Deep Learning OR Neural network OR Chatbot OR Reinforcement Learning) AND (Psychosis OR Schizo*) AND (Psychosocial function OR Social Function OR Social cognition OR Cognitive remediation OR Cognitive function OR Functional outcome OR Quality of life OR Vocational skill OR Psychotherapy). The search strings used were detailed in **Supplementary Data (Table 1)**. In the initial screening, duplicate studies were eliminated. The titles of remaining studies were then reviewed to identify relevant studies. Subsequently, the abstracts and full texts were assessed for the eligibility of the studies.

### 3.2 Inclusion and exclusion criteria

Studies were included if they applied AI techniques in psychosocial function; included participants with schizophrenia, schizoaffective disorder, or other psychotic disorders. Studies were excluded if they were not available in English, not apply AI technologies, not involve individuals with psychosis, not focus on psychosocial function, no full text available, or they were non-empirical studies such as review articles and commentaries.

### 3.3 Data extraction

The relevant information was extracted from each study and summarized into **Table 1**. The systematically extracted information included authors, country of origin, year of publication, sample size, outcome measures, AI techniques applied, and key findings. The process was carried out independently by two reviewers. To ensure consistency between reviewers, the review process was iteratively refined until a Cohen’s kappa coefficient (κ) of at least 0.85 was achieved.

**Table 1:** Characteristics of the included studies.

Author/ year/ country	Study design	Sample	Data	Model	Outcome measure	Study aim(s)	Key finding(s)
Supervised ML (n=11)							
Badel et al. (39) United State	Observational cross-sectional	154 SZ patients and 154 HC	Retrospective	SGD, AdaBoost, NN	SLOF, OSCARS, PANSS, BDI	To distinguish SZ patients from HC and predict outcomes of social functioning and social cognition	The ML models outperformed traditional statistical methods in the differentiation of SZ patients from HC The ML models demonstrated moderate to strong predictive power
Bosco et al. (40) Italy	Observational cross-sectional	22 patients with chronic SZ, 22 HC	Prospective	Bayesian network	T.h.o.m.a.s	To model ToM facet relationships between SZ patients and HC	SZ patients had lower probabilities of achieving high scores on all T.h.o.m.a.s. scales compared to HC The first-person ToM is foundational, influencing more complex ToM facets
de Nijs et al. (41) Netherlands	Observational cohort	523 patients with a diagnosis of psychotic disorders	Prospective	Linear SVM	GAF	To predict symptomatic remission and global functioning outcomes in 3- and 6-year	The ML model demonstrated modest predictive accuracy (Accuracy 62-68%) for long-term psychosis outcomes.
Li et al. (42) China	Observation cohort	550 SZ patients who were receiving atypical antipsychotics	Prospective	RF	PSP	To predict social functional improvement in SZ patients after 3 months of atypical antipsychotic monotherapy.	The RF model predicted significant social functional improvement with an accuracy of 79.5% and an AUC of 86.7%
Lin et al. (43) Taiwan	Observational cohort	302 Taiwanese with a diagnosis of SZ	Prospective	Bagging ensemble with either MFNNs or SVM	QLS, GAF	To predict functional outcomes by analyzing clinical symptoms and cognitive functions	The bagging ensemble model, which incorporated feature selection and targeted clinical symptom scales, demonstrated superior performance compared to other models in predicting functional outcomes.
Lin et al. (44) Taiwan	Observational cross-sectional	302 Taiwanese with a diagnosis of SZ	Retrospective	Bagging ensemble with linear regression	QLS, GAF	To predict functional outcomes based on their genetic profiles	The prediction performance of the bagging ensemble with feature selection surpassed that of the other models. Genetic biomarkers, when paired with AI, can enhance predictions of psychosocial functioning
Leighton et al. (45) England	Observational cohort	1027 patients aged 14–35 with FEP	Prospective	Logistic R	PANSS, GAF, EQ-5D, WHO, QOL	To predict 1-year outcomes on psychotic symptoms, social and vocational functioning and QoL) in FEP patients using baseline clinical data	Models predicted outcomes with AUCs of 0.70-0.74, indicating good predictive performance for 1-year outcomes
Shibata et al. (46) Japan	Observational cross-sectional (Prospective dataset)	18 patients with SZ, with a subset of 6 measured twice for longitudinal analysis	Prospective	k-NN, RF	J-SQLS (adapted Japanese version from QLS)	To estimate subjective QoL in schizophrenic patients by analyzing speech features	Both models can estimate QoL scores from speech features, with better performance in longitudinal settings.
Walter et al. (47) Germany	Multi-method: Observational cohort (for training cohort) & RCT (intervention sample)	70 SZ patients in the training cohort; 54 SZ patients in the testing sample	Prospective (training cohort)	SVM	GF-S	To predict social functioning using baseline cognitive data To evaluate the impact of SCT on these predictions	Stronger performance in the training cohort than in the intervention sample SCT led to a statistically significant improvement in predicted social functioning compared to TAU
Wang et al. (48) United State	Observational cohort	55 individuals with SZ	Prospective	RF, extremely randomized trees, XGBoost, SVR, Linear Regression	SFS	To predict social functioning (SFS scores) from mobile sensing data captured from a mobile app (CrossCheck)	The ML models achieved moderate to good predictive accuracy, with the weakest performance in the recreation sub-scale and the strongest in the functional independence sub-scale
Supervised ML + Unsupervised ML (n=1)							
Miley et al. (49) United State	Observational cohort	76 out-patients with SZ	Prospective	RF, Logistic regression	ER40, PROID, PFMT, MSCEIT, EA, UPSA-2, GFS, QLS, SLOF, SFS, TEPS, WTAR	To identify distinct response patterns to social cognition training in schizophrenia	Moderate predictive ability in predicting membership in the benefiting group A better baseline ToM was more likely to benefit from training
AutoML (n=1)							
Lin et al. (50) Taiwan	Observational cross-sectional	302	Prospective	TPOT	MSCEIT	To predict social cognition in schizophrenia using neurocognitive data and clinical variables	The best-performing model (Model-12) used 7 factors (age, gender, and five neurocognitive domains)
Causal Machine Learning (n=1)							
Miley et al. (51) United State	Observational cohort	276 individuals with early SZ	Retrospective	GFCI	QLS	To identify high-impact treatment targets using causal discovery analysis	Social affective capacity and motivation directly influenced the causes of social and occupational functioning, whereas cognitive impairment and DUP were not found to be direct causes.
DL, RL & NLP (n=1)							
Lin et al. (52) United State	Non-observational (Proof of Concept study)	Not specified	Retrospective	DDPG, TD3, BCQ	Working Alliance Inventory	To provide real-time recommendations on conversation topics during psychotherapy sessions, tailored to specific psychiatric conditions and therapeutic objectives	Moderate accuracy in recommending conversation topics Offline reinforcement learning (BCQ) showed better generalization across disorders

AUC, Area Under the Receiver Operating Characteristic Curve; BCQ, Batch Constrained Q-Learning; BDI, Beck Depression Inventory; DDPG, Deep Deterministic Policy Gradient; DUP, Duration of Untreated Psychosis; EA, Empathic Accuracy Task; ER40, Penn Emotion Recognition Task; FEP, First Episode Psychosis; GAF, Global Assessment of Functioning; GFCI, Greedy Fast Causal Inference; GF-S, Global Functioning-Social; HC, Healthy Control; J-SQLS, Japanese Schizophrenia Quality of Life Scale; k-NN, k-Nearest Neighbor; MFNN, Multilayer Feedforward Neural Network; MSCEIT, Mayer-Salovey-Caruso Emotional Intelligence Test; NN, Neural Network; OSCARS, Observational Social Cognition: A Rating Scale; PANSS, Positive and Negative Syndrome Scale; PFMT, Penn Faces Memory Test; PROID, Prosody Identification; PSP, Personal and Social Performance; QLS, Quality of Life Scale; RF, Random Forest; SCT, Social Cognition Training; SFS, Social Functioning Scale; SGD, Stochastic Gradient Descent; SLOF, Specific Levels of Functioning Scale; SVR, Support Vector Regression; SVM, Support Vector Machine; TAU, Treatment as Usual; TD3, Twin Delayed Deep Deterministic Policy Gradient; TEPS, Temporal Experience of Pleasure Scale; T.h.o.m.a.s, Theory of Mind Assessment Scale; TPOT, Tree-based Pipeline Optimization Tool; UPSA-2, UCSD Performance-based Skills Assessment (Shortened Version); WTAR, Wechsler Test of Adult Reading

### 3.4 Quality assessment

To ensure methodological rigor aligned with study design, a tailored quality assessment approach was adopted. The Newcastle-Ottawa Scale (NOS) and its adapted version were used to appraise observational cohort and cross-sectional studies respectively. The Cochrane Risk of Bias Tool (RoB 2.0) was applied to the randomized controlled trial (RCT). For non-observational predictive modeling studies, the PROBAST (Prediction Model Risk of Bias Assessment Tool) was employed. This strategy ensures that each study was evaluated using the most appropriate and context-relevant criteria. The Grading of Recommendations Assessment, Development, and Evaluation (GRADE) was used to evaluate the quality of the collected evidence. The quality ratings were conducted independently by two reviewers.

### 3.5 Statistical analysis

Performance metrics from the studies included in the analysis were extracted for quantitative synthesis. Only metrics that appeared in at least two independent studies were included in the meta-analysis. Metric values from external validation of the studies were prioritized. The evaluation of potential heterogeneity among studies was conducted using Cochran’s Q test, with results expressed as the I² statistic. A random-effects model was used to compute pooled estimates and their 95% confidence intervals. To investigate the sources of heterogeneity, subgroup analyses were performed. The robustness of the pooled estimates was assessed through sensitivity analyses by adopting the leave-one-out (LOO) approach. Publication bias was assessed using funnel plots and Egger’s regression tests, which was only applied when there were at least ten independent estimates to maintain statistical validity. Funnel plots were examined descriptively to identify potential asymmetry. All statistical analyses were carried out using R software (Version 4.5.0).

## 4 Result

### 4.1 Study selection & characteristics

**Figure 1:**
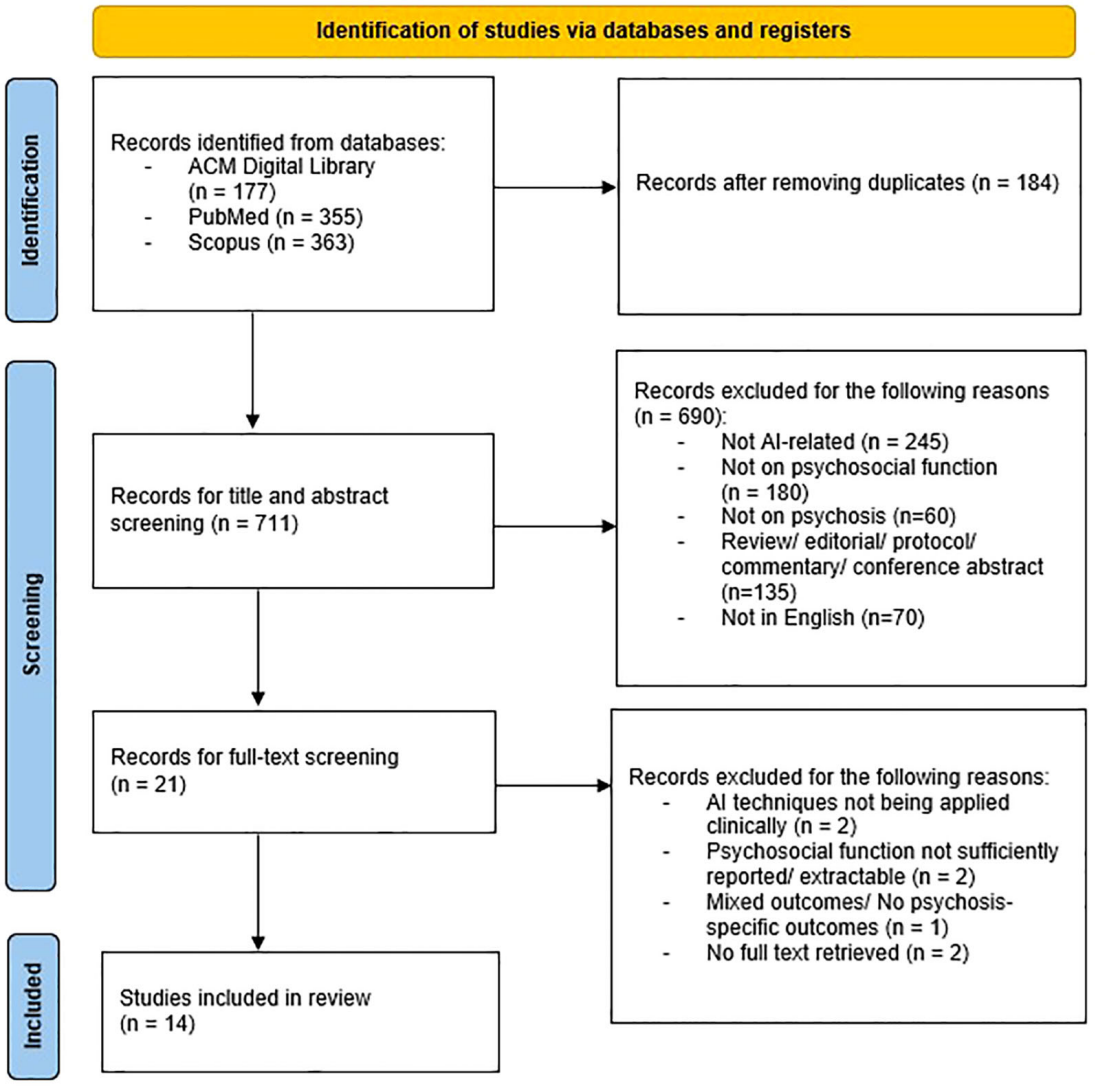
PRISMA (preferred reporting items for systematic reviews and meta-analyzes) flow diagram.

From the initial literature search, studies were identified. Once duplicates were removed, 711 studies remained that were deemed potentially relevant. After screening titles and abstracts, 690 studies were excluded because they did not meet the inclusion criteria. Subsequently, 37 studies were included after full-text analysis. A total of 14 studies that fully met the inclusion criteria were selected in this review. Of these, 9 studies were conducted in Western countries and 5 studies were conducted in Asian countries. **Figure 1** shows the PRISMA flow diagram of the search. It was noted that 3 papers were authored by conducted by a research team from Taiwan. All of which utilized the same group of subjects but different topic focuses and data types.

### 4.2 Characteristics of AI methodology

**Figure 2:**
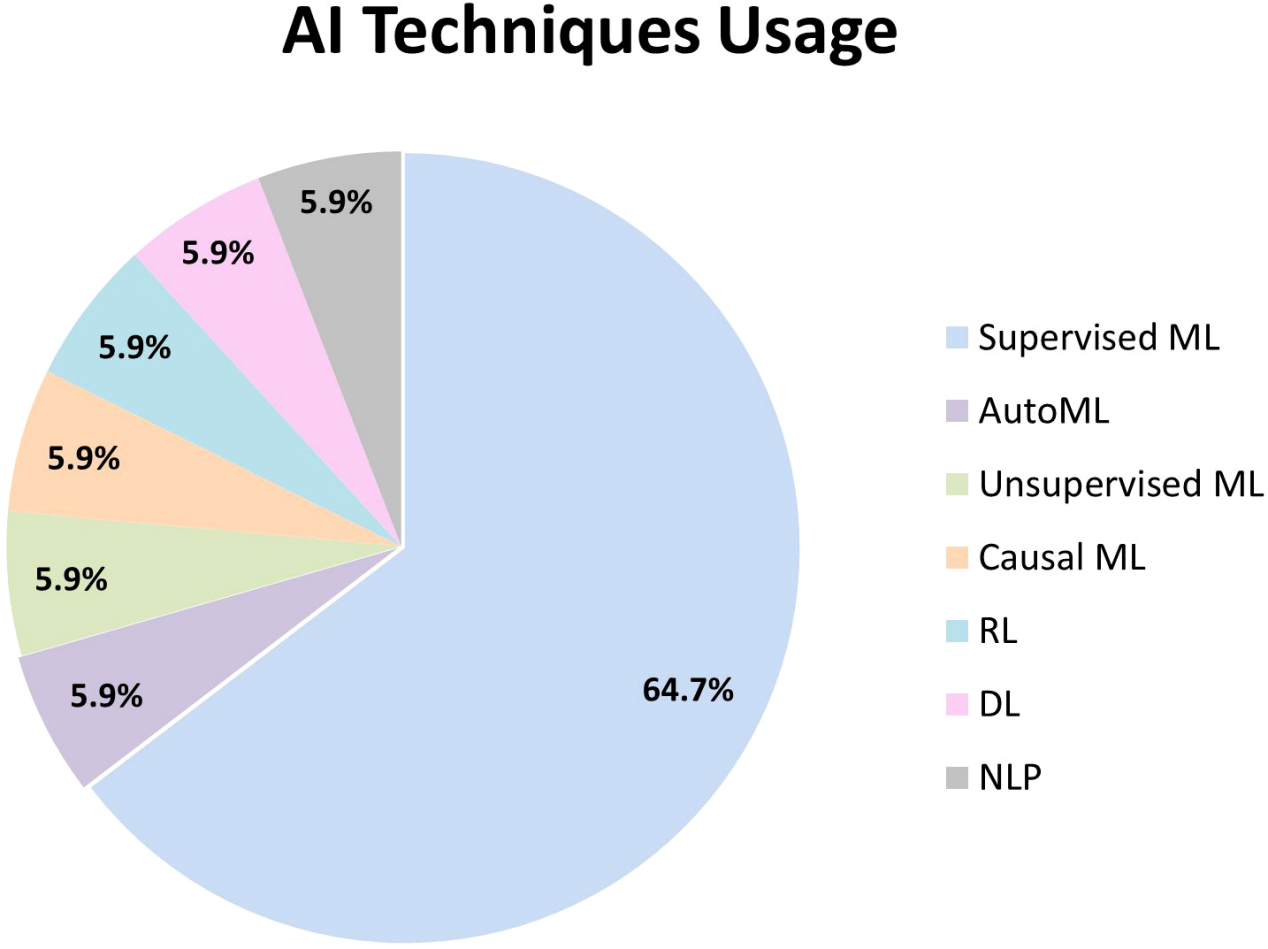
AI techniques usage.

The included studies applied a diverse range of AI techniques, namely supervised ML, unsupervised ML, causal ML, RL, DL, AutoML, and NLP (**Figure 2**). While one study used both unsupervised and supervised ML, another one combined RL, DL, and NLP in its AI system.

Supervised ML was predominantly utilized within the psychosis and schizophrenia population (39–49). Random forest (RF) was the most frequently utilized model (42, 46, 48, 49). The following widely used model was support vector machines (SVM), which were employed in three studies (41, 47, 48). Other commonly adopted supervised models included linear regression (44, 48), bagging ensemble (43, 44) and boosting techniques – AdaBoost (39) and XGBoost (48). Regularized regression models, including elastic net (45), Least Absolute Shrinkage and Selection Operator (LASSO) (42), neural networks (39), k-nearest neighbors (k-NN) (46), and Bayesian networks (40) were applied in individual studies as alternative supervised models. Feature selection techniques were incorporated in three studies to enhance model performance by selecting the most predictive variables, including M5 Prime (43, 44), LASSO (42), and Recursive Feature Elimination (RFE) (41).

Unsupervised ML with the use of latent class growth analysis (49), AutoML with Tree-based Pipeline Optimization Tool (TPOT) (50), and causal ML with Greedy Fast Causal Inference (GFCI) algorithm (51) were employed in single studies. One study combined RL, DL, and NLP, utilizing algorithms such as Deep Deterministic Policy Gradients (DDPG), Twin Delayed DDPG (TD3), and Batch Constrained Q-Learning (BCQ), along with Doc2Vec, to develop its AI system (52).

### 4.3 Application of AI on psychosocial domains

While 9 studies focused on applying AI techniques to specific psychosocial aspects, 5 studies covered more than one domain in their application. Three main psychosocial domains were identified: social and occupational functioning, social cognition, and QoL.

#### 4.3.1 Social and occupational functioning

Social and occupational functioning was the most researched domain (n=10), in which most studies considered functional levels and employment status as indicators of overall social recovery. Among the 10 studies, 4 studies used the Global Assessment of Functioning (GAF) as the outcome measurement tool. Ensemble learning methods, specifically RF with feature selection and bagging, were found to be the most outstanding supervised ML models in predicting outcomes, highlighting their high predictive ability with low error rates (42, 43).

#### 4.3.2 Social cognition

4 studies focused on social cognition (39, 40, 49, 50), with one specifically investigating the ToM within the schizophrenia population (40). By embedding Bayesian networks in supervised ML, it demonstrated an outstanding performance with high accuracy in differentiating individuals with schizophrenia from healthy participants purely based on their ToM abilities on T.h.o.m.a.s, exploring hierarchical relationships among ToM facets, and identifying the most pronounced ToM deficits within schizophrenia (40). To serve a similar purpose, Badal et al. (39) achieved good to excellent ability in differentiation based on two emotion recognition tasks (BLERT & ER40), by using Gini Importance – a feature selection method in supervised ML. The use of AI techniques in classification and understanding of the complexity of social cognition is therefore emphasized.

#### 4.3.3 Quality of life

All 4 studies used supervised ML for the prediction of QoL (43–46). Bagging ensemble with feature selection again was the highest performing AI method, with high accuracy in forecasting QoL in individual with schizophrenia based on cognitive abilities and clinical symptoms (43). All 4 studies employed dedicated QoL measures, in which QLS was frequently used (43, 44, 46).

### 4.4 Performance of AI applications

Across the studies, the application of AI can be categorized into three main areas: predicting psychosocial outcomes, mechanistic exploration of psychosocial function in psychosis, and providing therapeutic support in psychosocial interventions. Various metrics were utilized to assess the model’s performance. **Table 2** describes the metrics adopted and the performance range among included studies.

**Table 2:** Metrics adopted and performance range.

Metric	Use of metric	Interpretation	Performance range in the included studies
Discrimination & classification metrics			
AUC	To evaluate a model’s discriminatory power	Higher values indicate better discrimination between classes. Values ≥0.80 generally good; 0.70–0.80 acceptable.	0.56-0.867
F1 Score	To assess predictive performance by balancing precision and recall	Closer to 1 is better; values ≥0.80 denote strong performance.	0.74-0.85
Accuracy	To measure the overall proportion of correct predictions	Higher is better; ≥0.80 is typically good.	64%-86.4%
Balanced Accuracy (BAC)	To evaluate model performance on imbalanced datasets	Values >0.50 indicate better-than-chance; ≥0.70 is often acceptable.	59.3%
Error & regression metrics			
RMSE	To quantify prediction errors	Lower values indicate a more minor prediction error	6.43-9.7
MAE	To quantify average absolute prediction error	Lower values indicate a minor error	2.17–2.79
Explanatory power			
Proportion of Variance Explained (PVE)	To quantify how well a model captures the patterns and variability within the data	Higher values (closer to 1) indicate more variability explained; values <0.30 indicate low explanatory power.	0.2
Effect Size	To quantify the degree of association between two variables or a difference between groups	Larger absolute values indicate more substantial effects; 0.20=small, 0.50=medium, ≥0.80=large.	0.77-1.5
Structural equation model (SEM) fit			
Comparative Fit Index (CFI)	To assess model fit	Values ≥0.90 indicate acceptable model fit; ≥0.95 ideal; <0.90 suboptimal.	0.884
RMSEA	To assess model fit, accounting for model complexity	<0.05 good, 0.05–0.08 acceptable, >0.10 poor; lower is better.	0.066

#### 4.4.1 Prediction of psychosocial outcomes

11 out of 14 studies focused on predicting psychosocial outcomes, including social functioning, QoL, social cognition, and global functioning (39, 41–50). Various data types, including clinical, genetic, cognitive, and behavioral data, were utilized to develop AI models with varying predictive success. Some studies demonstrated robust performance; for example, Badal et al. (39) achieved F1 scores of 0.74–0.81 in predicting social and social cognitive functioning. Similarly, Li et al. (42) reported an AUC of 0.867 and an accuracy of 79.5% in forecasting social functional improvement. Furthermore, Lin et al. (43) showcased the lowest RMSE of 6.43 when using RF to predict QoL.

However, the performance was modest in other studies. Leighton et al. (45) reported AUCs ranging from 0.703 to 0.736 for forecasting outcomes like symptom remission and vocational recovery. Walter et al. (47) reported a BAC of 59.3% for social functioning, with a 19.6% decline from the training to the testing phase. Likewise, Wang et al. (48) reported MAE values of 2.17-2.79, indicating approximately 10% of prediction error in predicting the score of the Social Functioning Scale. Moreover, low explanatory power in the variance of social cognition was reflected with a PVE of 0.2 (50).

#### 4.4.2 Mechanistic exploration of psychosocial function

Two studies employed AI to explore the underlying mechanisms of psychosocial functioning. Miley et al. (51) yielded strong effect sizes (0.77–1.5) in examining the interplay between motivation, social affective capacity, and functional outcomes in early schizophrenia; however, they reported suboptimal model fit (CFI, 0.884, RMSEA, 0.066). While Bosco et al. (40) achieved an accuracy of 86.4% in differentiating patients from controls, they also provided insights into the hierarchical structure of ToM facets. Similarly, Badal et al. (39) achieved AUCs of 0.83–0.86 and F1 scores of 0.81–0.85 in differentiating between individuals with schizophrenia and healthy controls using facial affect recognition data, demonstrating good to excellent performance.

#### 4.4.3 Therapeutic support in psychosocial interventions

Lin et al. (52) examined the role of AI in supporting psychosocial interventions. It employed real-time recommendation systems during psychotherapy sessions. This model attained a moderate accuracy of 64% relative to human therapist decisions, indicating a promising yet underdeveloped application.

#### 4.4.4 External validation

Of the four studies that conducted external validation to assess model generalizability beyond their training datasets, a decline in performance was consistently observed (41, 45, 47, 51). Potential overfitting to training data was found to be a common challenge. The magnitude of the performance drop varied: de Nijs et al. (41) showed a minimal accuracy decrease of 2.3%, indicating satisfactory generalizability in the prediction model for three- and six-year psychosocial outcomes, while Walter et al. (47) reported a substantial reduction in balanced accuracy from 78.9% to 59.3%, suggesting limited applicability to new populations. Outcome-specific trends were observed, with vocational recovery maintaining strong predictive power across datasets, whereas quality of life exhibited weaker external performance (45).

### 4.5 Meta-analysis of performance metrics – AUC

#### 4.5.1 Pooled AUC

**Figure 3:**
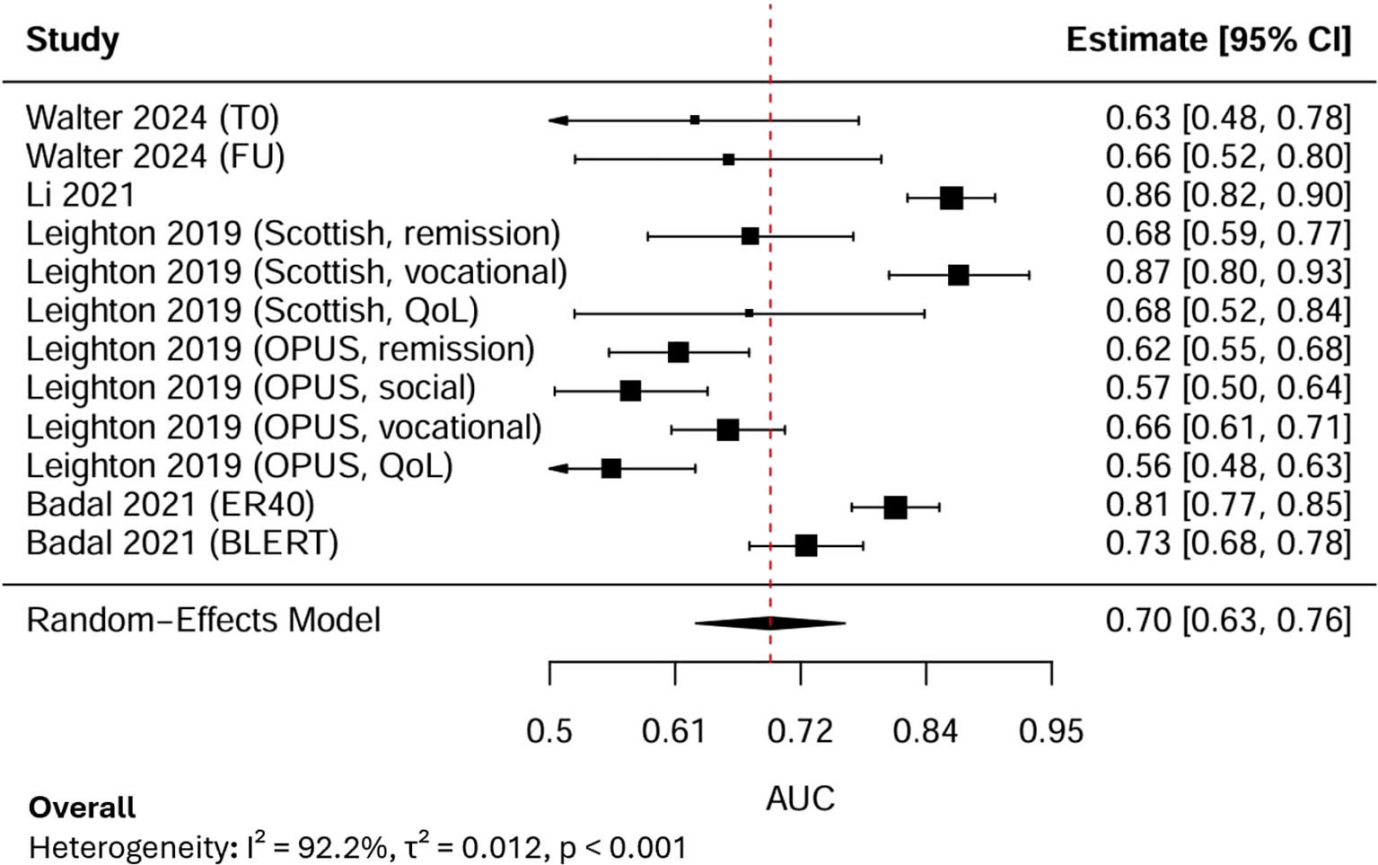
Forest plot for the pooled AUC.

12 AUC estimates from 4 independent studies were subjected to a random-effects meta-analysis (**Figure 3**). Each AUC estimate reported the predictive performance of AI models for psychosocial functioning in psychosis. The pooled AUC was 0.70, with the 95% Confidence Interval (CI) at 0.63-0.76, indicating moderate discriminative performance. Between-study heterogeneity was extremely high (I², 92.2%), reflecting substantial variability across outcome domains, sample sizes, and modelling approaches.

#### 4.5.2 Subgroup analysis of AUC

**Figure 4:**
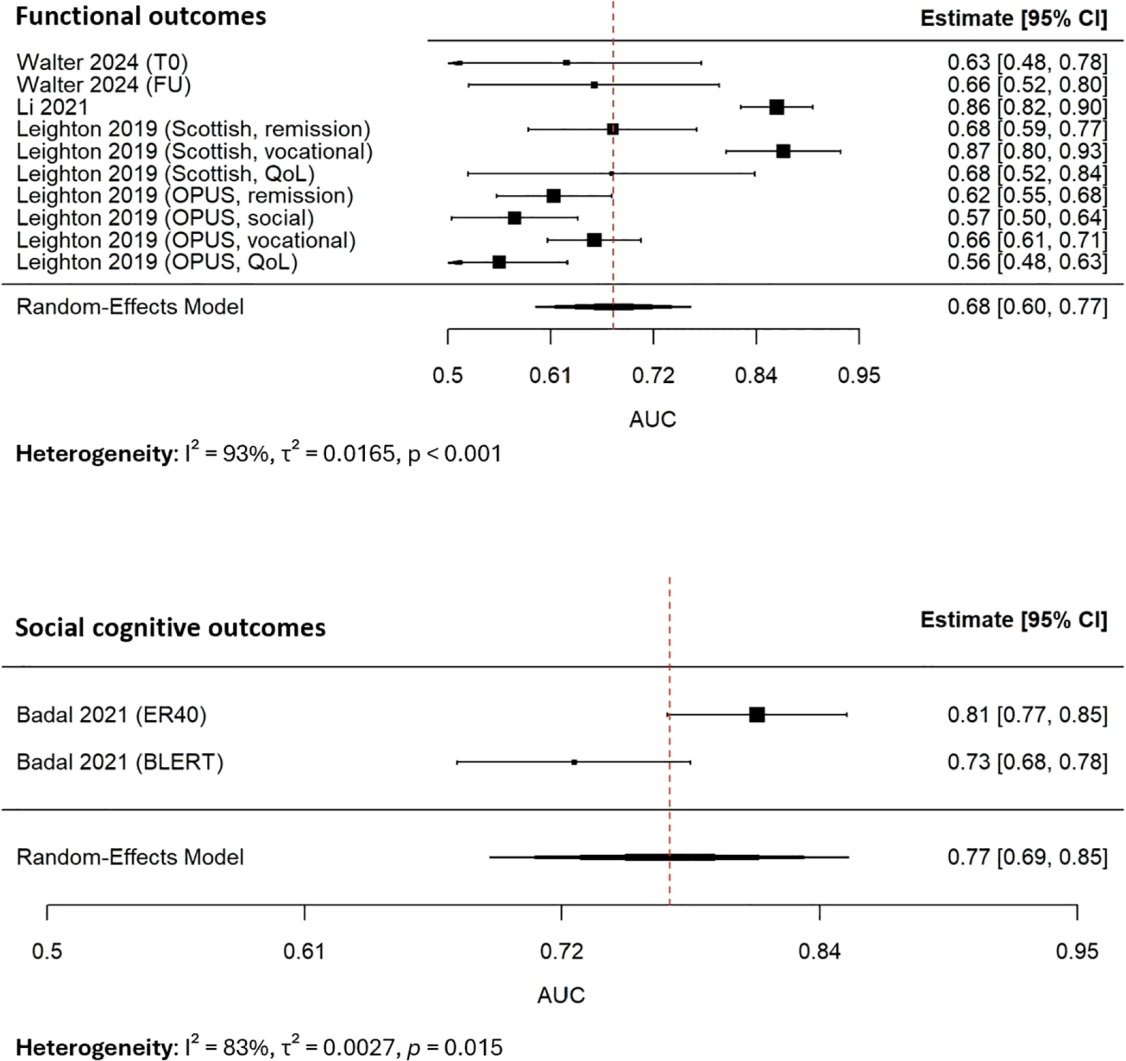
Forest plot for the subgroup analysis of AUC.

Subgroup analyses revealed systematic differences across outcome types (**Figure 4**). Models predicting functional outcomes (k=10) yielded a pooled AUC of 0.68 (95% CI: 0.60–0.77; I², 93%), while those applied to social cognition (k=2) achieved higher performance (0.77, 95% CI: 0.69–0.85; I², 83%). A test of subgroup differences using Cochran’s Q statistic indicated a statistically significant difference between functional outcome models and social cognitive outcome models (Q(between), 9.17, degrees of freedom, 1, p, 0.003). It suggested that model performance depended strongly on the outcome domain, with higher discriminative accuracy for facial emotion recognition and social cognition than for functional outcomes such as vocational and social skills.

#### 4.5.3 Sensitivity analysis of AUC

LOO sensitivity analysis demonstrated that sequentially excluding each study produced stable pooled AUC estimates ranging from 0.68 to 0.71, and all Cis overlapped with the overall pooled estimate (AUC, 0.70, 95% CI: 0.63–0.76). Although slight changes in heterogeneity were observed (I² reduced from 92% to approximately 90%), the variation remained statistically significant (p < 0.001). By specifically excluding the only study with a high risk of bias, the pooled AUC increased slightly to 0.71 (95% CI: 0.64–0.78), and heterogeneity decreased marginally (I², 91%, τ², 0.010, p < 0.001). These results showed that the overall findings were robust and not driven by the high-risk study, with the observed variability likely reflecting methodological and outcome-related differences across studies rather than bias from any individual source.

### 4.6 Meta-analysis of performance metrics - RMSE

#### 4.6.1 Pooled RMSE

**Figure 5:**
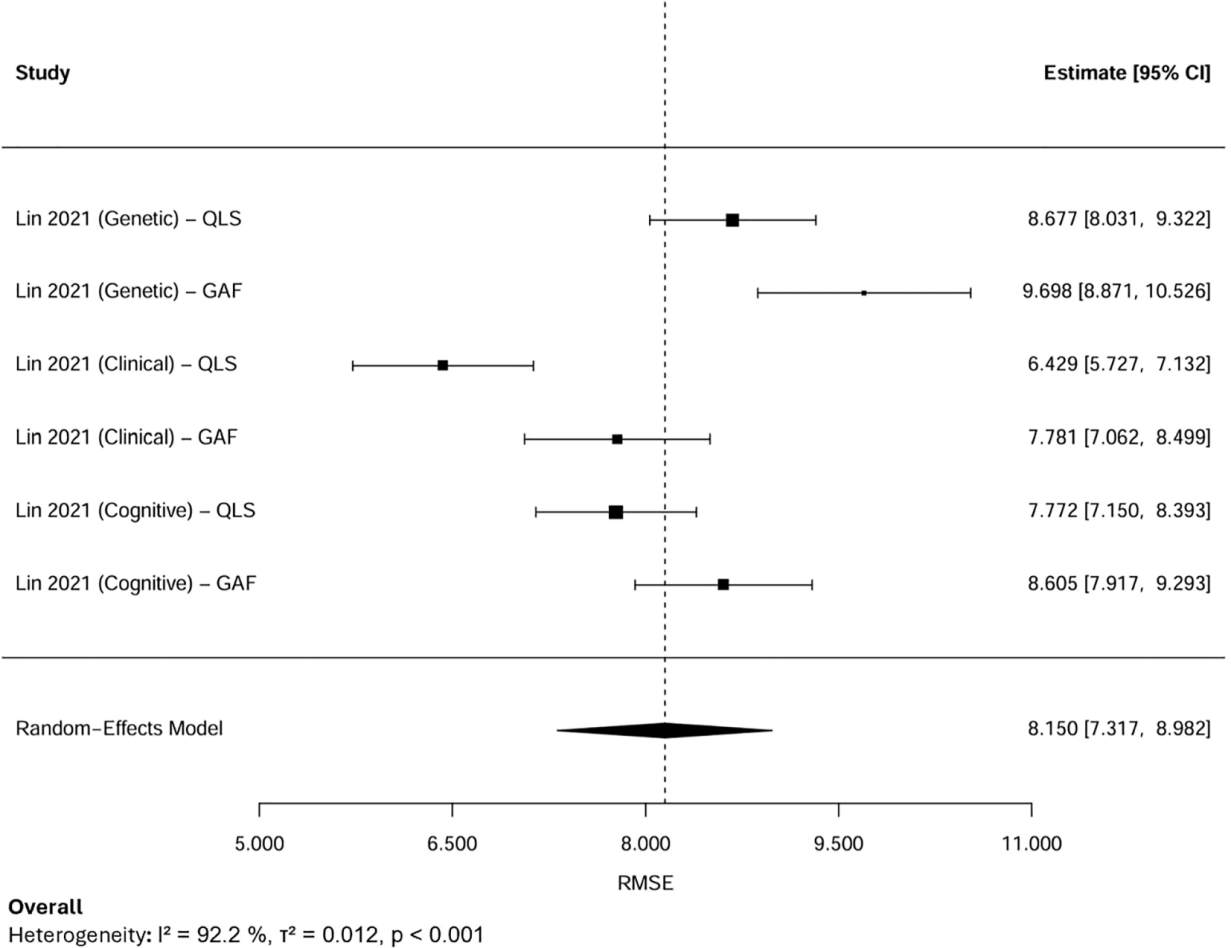
Forest plot for the pooled RMSE.

A random-effects meta-analysis was performed by aggregating six RMSE estimates from two studies that focused on the same cohort in Taiwan (n=302) (**Figure 5**). Because these estimates were derived from overlapping samples, the results were interpreted cautiously, acknowledging the potential for double-counting. The observed variability in RMSE values was therefore more plausibly attributed to differences in modeling strategies, predictor sets, and analytical approaches rather than to actual differences within the study population. The pooled RMSE was 8.15 (95% CI: 7.32–8.98), suggesting moderate predictive performance. Substantial heterogeneity was observed (I², 92.2%, τ², 0.012, p < 0.001).

#### 4.6.2 Subgroup analysis of RMSE

**Figure 6:**
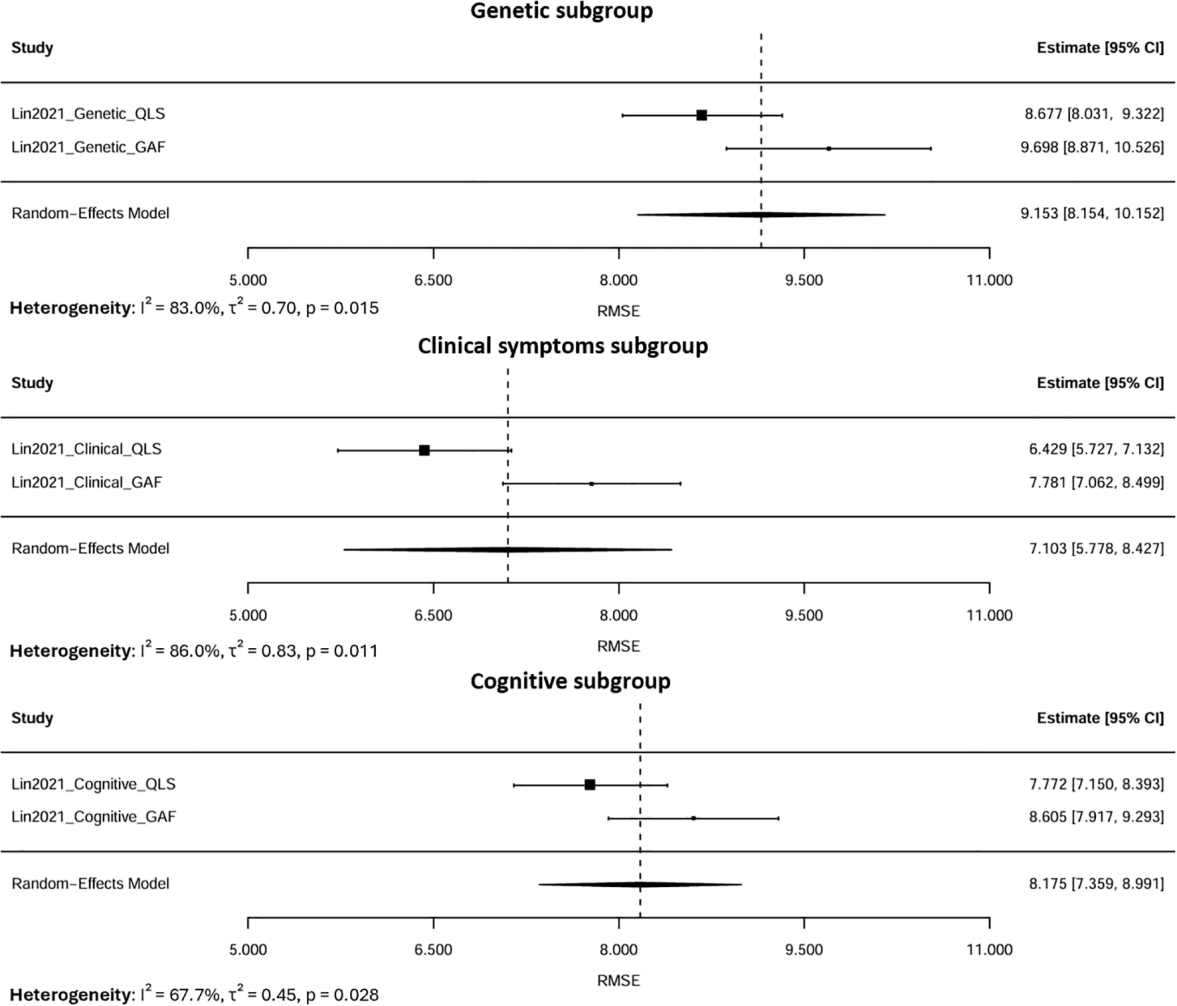
Subgroup meta-analysis of pooled RMSE by predictor domains (Genetic, Clinical, Cognitive).

Subgroup analyses were conducted based on predictor domains (**Figure 6**). When stratified by predictor domain, clinical symptom-based models produced the lowest pooled RMSE of 7.10 (95% CI: 5.78–8.43), followed by cognitive models at 8.17 (95% CI: 7.36–8.99). Conversely, genetic predictor models yielded the highest RMSE of 9.19 (95% CI: 7.96–10.41), indicating the highest error.

Heterogeneity within these subgroups was lower than that observed in the overall pooled analysis (I², 67.7–86% *vs*. 92.2%), suggesting that stratifying by predictor domain partially accounted for between-model variability. The remaining heterogeneity likely reflected differences in modeling approaches, predictor characteristics, and methods of feature selection rather than random errors. Since all estimates were derived from the same cohort, the residual variability represented within-sample differences in model performance rather than actual inconsistencies between studies. Overall, these results indicated that models based on clinical symptom features exhibited superior predictive accuracy, whereas those relying solely on genetic data performed less effectively for modeling psychosocial functioning in this cohort.

#### 4.6.3 Sensitivity analysis of RMSE

LOO sensitivity analysis showed that the pooled RMSE remained stable after sequentially excluding each estimate. The pooled values ranged narrowly from 7.86 to 8.48, and all CIs overlapped with the overall estimate, indicating that no single model substantially influenced the pooled estimate.

### 4.7 Publication bias

**Figure 7:**
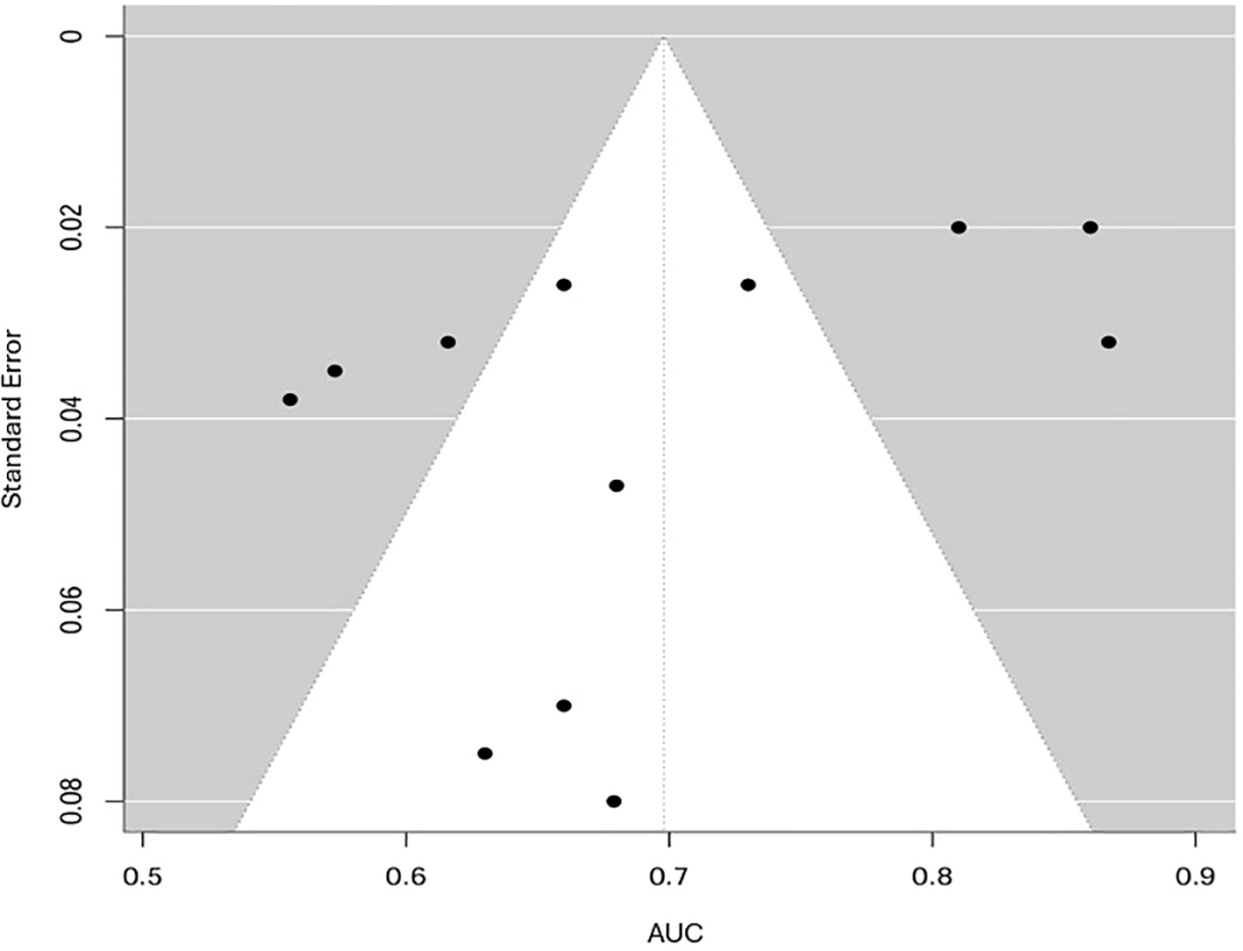
Funnel plot for the meta-analysis of AUC performance.

To evaluate potential publication bias, funnel plots for both AUC and RMSE were analyzed. The funnel plot for AUC (**Figure 7**) displayed slight asymmetry; however, Egger’s test results (p, 0.08) did not demonstrate significant small-study effects, indicating a low likelihood of publication bias. Given the restricted number of independent studies and the high heterogeneity (I² > 90%), the observed asymmetry was more likely due to methodological and clinical diversity rather than selective reporting. Consequently, the funnel plot should be interpreted descriptively rather than as definitive evidence of bias.

**Figure 8:**
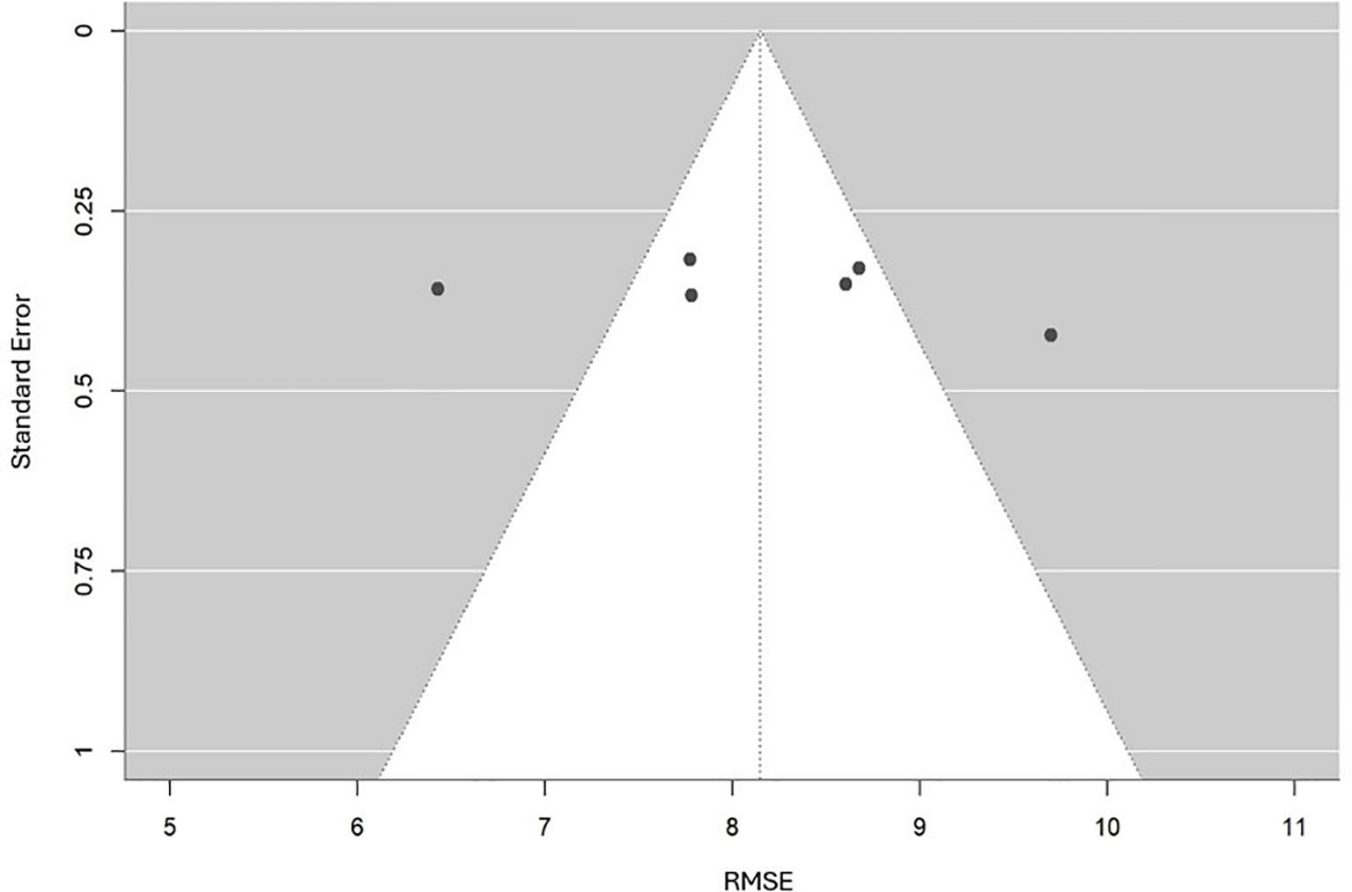
Funnel plot for the meta-analysis of RMSE performance.

For RMSE, Egger’s regression test was not conducted because only six estimates were available, all sourced from the same Taiwanese cohort, which violated the assumption of statistical independence. A descriptive funnel plot (**Figure 8**) was created to illustrate the distribution of RMSE values in relation to their standard errors. This plot exhibited a generally symmetrical pattern around the pooled estimate, with no clear signs of bias. However, since all estimates were derived from the same dataset, this apparent symmetry should not be considered conclusive evidence against publication bias. Instead, the funnel plot served as a visual summary of estimate precision and variability, indicating that the observed differences are likely due to methodological variations across models rather than selective reporting.

### 4.8 Quality of evidence

#### 4.8.1 Quality assessment

**Figure 9:**
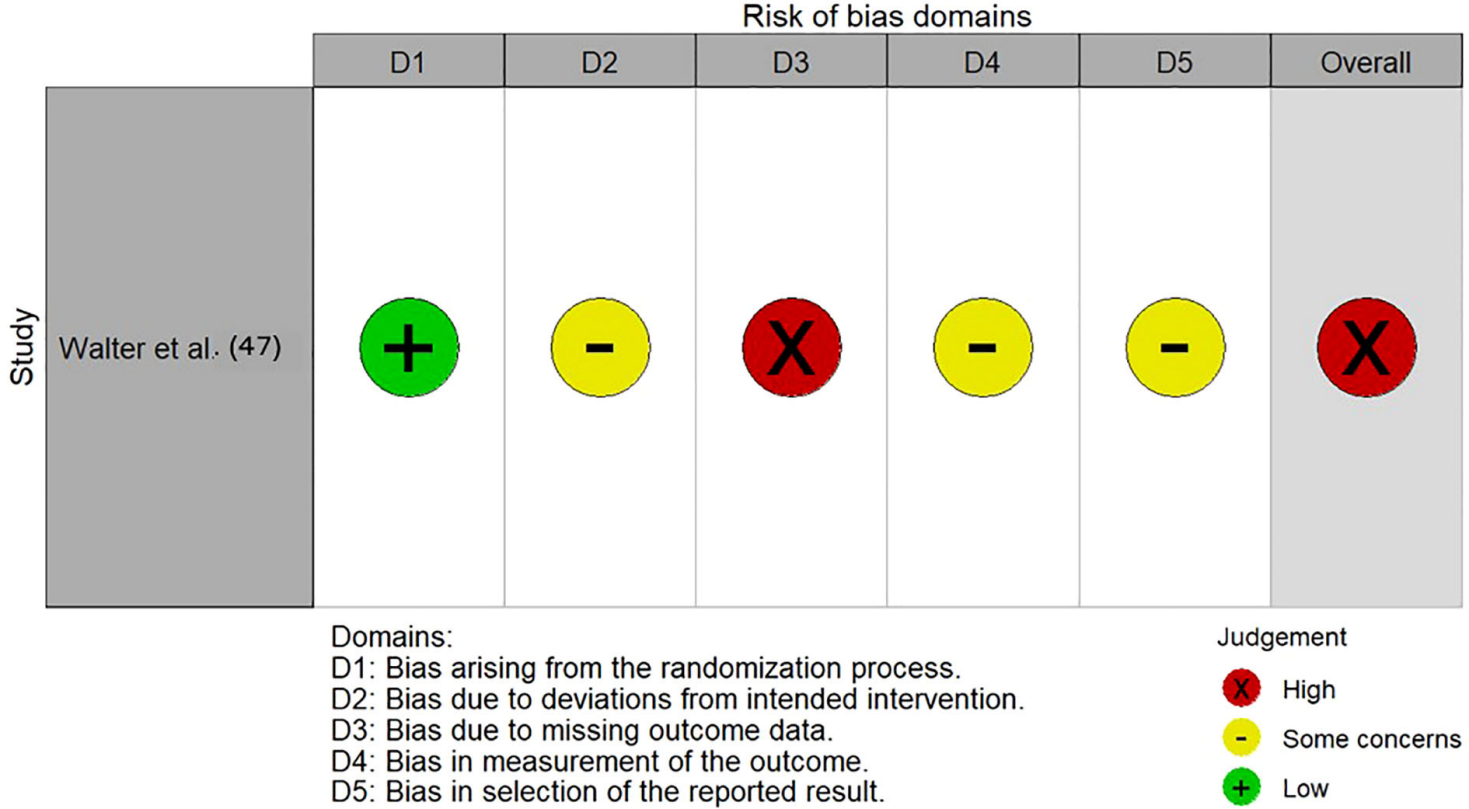
Risk of bias traffic-light plot for RCTs.

**Figure 10:**
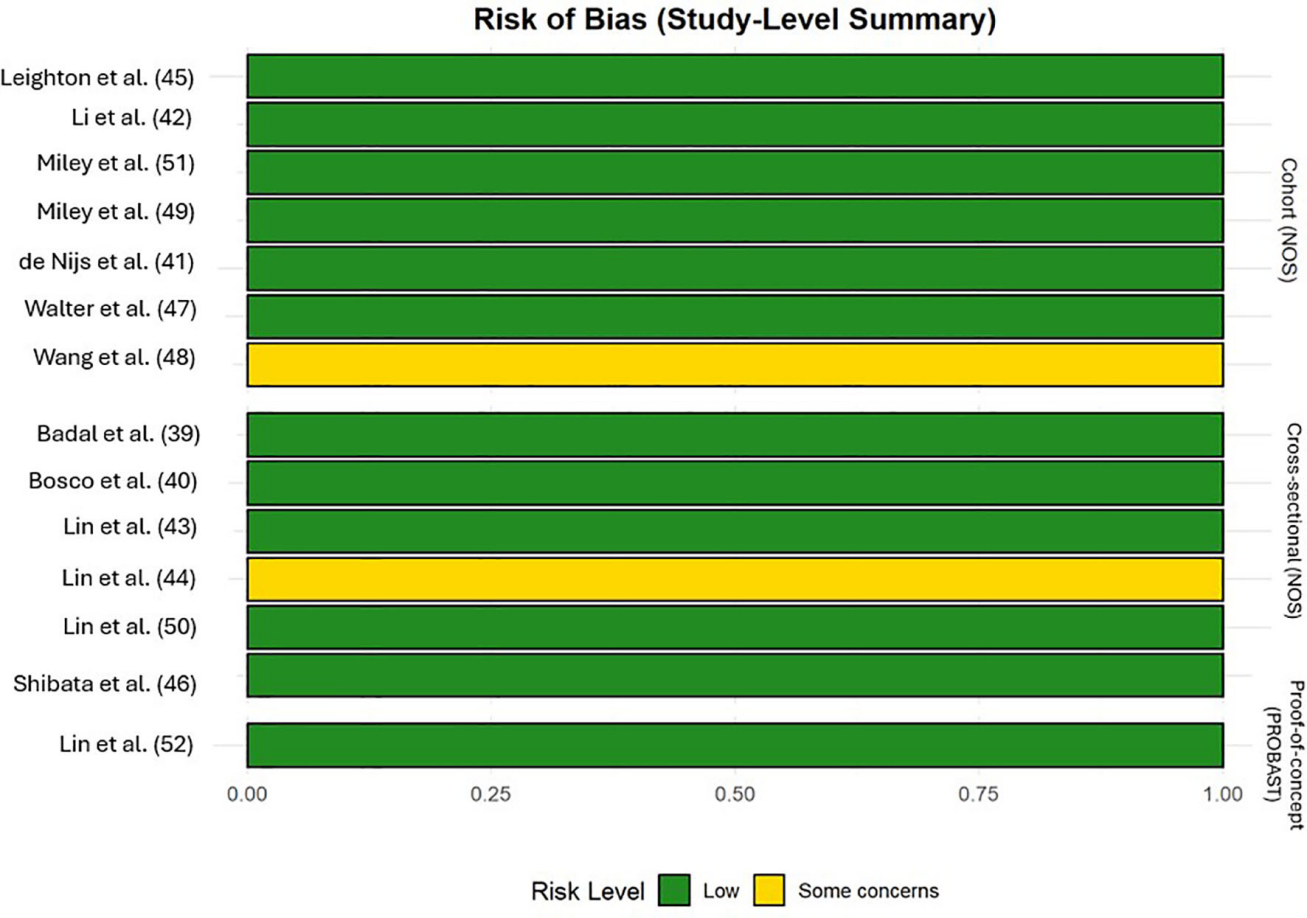
Summary bar of risk of bias across cohort, cross-sectional and proof-of-concept studies.

The majority of included studies demonstrated low risk of bias, suggesting good methodological quality and overall robustness of the synthesized findings. Two studies (45, 48) were rated as fair quality, reflecting some concerns, primarily regarding representativeness and sample size. The single RCT (47) showed a high overall risk of bias due to missing outcome data, whereas the prediction model study (52) was rated as low risk across all PROBAST domains. **Figures 9**, **10**, **Supplementary Data (Table 2)** illustrated the results of the quality assessment.

#### 4.8.2 GRADE

For meta-analyzed performance metrics (AUC and RMSE), the overall evidence was evaluated using the GRADE approach. Certainty of evidence was assessed using GRADE across five domains: risk of bias, inconsistency, indirectness, imprecision, and publication bias. Because the included studies were predominantly observational, the starting level was rated as ‘low’. The certainty for AUC was downgraded for inconsistency (I², 92%) and indirectness (heterogeneous outcomes and methods), while RMSE was further downgraded for imprecision due to reliance on a single cohort, resulting in overall low and very-low certainty ratings, respectively. **Supplementary Data (Table 3)** provided the corresponding GRADE summary of findings table.

## 5 Discussion

### 5.1 Principal findings

To our knowledge, this is the first systematic review and meta-analysis focusing on the application of AI in psychosocial function in psychosis. The 14 eligible studies highlighted the diverse use of AI techniques, predominantly using supervised ML, alongside unsupervised ML, causal ML, DRL, and AutoML. Our findings from this review aligned with another systematic review on AI targeting the schizophrenia population by Yang et al. (36) which also identified supervised ML as the most prevalent approach. Our findings were also coherent with the projection, which anticipated that ML will become the major share of the AI market in healthcare (16). However, compared with the broader application of AI in mental health, a more balanced spread across diverse technologies was evident, such as conversational AI for therapeutic support, computer vision for emotion recognition, and AI-enhanced virtual reality for immersive therapy in psychiatric disorders (53). This contrast implied that the utilization of AI in psychosis remains in its early stages, with a limited scope that primarily emphasizes ML over the broader range of AI innovations seen in other mental health domains. Three key areas of application in psychosocial function were identified, including outcome prediction, mechanistic exploration, and therapeutic support, with prediction being the most common primary task undertaken. Psychosocial domains, including social functioning, occupational functioning, social cognition, and QoL were covered.

The findings from our meta-analyses, which were based on the most commonly adopted performance metrics, indicated moderate discriminative and predictive accuracies of AI models in addressing psychosocial functioning in psychosis. Notably, subgroup analyses indicated superior performance in predicting social cognition than broader functional abilities. Also, models using psychiatric symptoms as the predictor was found to have highest predictive accuracy when compared with models with using cognitive function and genetic factors as predictors. The heterogeneity (I², 67.7% to 93%) across meta-analyses underscored multifaceted factors including varying sample sizes, divergent outcome domains, disparate modelling approach. Rather than reflecting random error or systematic bias from individual studies, this variability likely stemmed from methodological diversity and non-standardized analytic practices across models. These findings emphasized the significance of adopting standardized modeling protocols, integrating multimodal data, and including diverse cohorts to enhance the robustness and generalizability of AI-based psychosocial care in psychosis.

### 5.2 Integration of AI into clinical practice

#### 5.2.1 Early identification of at-risk populations

Predictive models, particularly ensemble learning methods, demonstrated the ability to predict social and occupational function in psychosis accurately (42, 44). Instead of relying on a single model, ensemble methods train several models whose predictions were combined to improve overall accuracy and robustness. In this review, the RF and bagging ensemble specifically demonstrated outstanding predictive ability in handling complex datasets with non-linear relationships, such as those related to social functioning. Moreover, it was found that both models utilized feature selection methods, such as LASSO and M5 Prime, which excelled at selecting impactful features, thereby further enhancing the accuracy and reliability of the results. The predictive modeling showed promise in identifying individuals at risk of poor social functioning and social cognition, enabling early interventions such as social cognition and interaction training (39, 42). The finding was parallel to that of Rotenberg et al. (54), which also found that the RF model outperformed other AI approaches in clinical prediction. Nonetheless, two reviews found no superiority of AI approaches in predicting clinical outcomes (55, 56). The inconsistencies in the findings regarding the superiority of AI approaches highlighted the necessity for further research, particularly in evaluating and contrasting various models to optimize model selection in clinical applications.

Apart from facilitating the early identification of psychosocial outcomes, the excellent discriminatory ability between schizophrenia and healthy controls, as indicated by evaluation of accuracy, confidence ratings, and reaction times on facial emotion recognition tasks, suggested potential in detecting schizophrenia (51). According to Lai et al. (30), the majority of papers focused on using brain imaging data from MRI scans, PET scans, and EEG for AI-augmented detection and classification. The higher accessibility and cost-effectiveness of obtaining behavioral data compared to brain imaging techniques may broaden their applicability for the widespread detection of at-risk populations. However, given the consistent findings of high predictive ability with brain imaging data (42, 57–61), it may serve as a complementary role with neuroimaging, offering a more holistic understanding of schizophrenia.

#### 5.2.2 Data-driven assessment & continuous monitoring

Moreover, AI holds significant promise for enabling objective, data-driven, and continuous assessment monitoring, which is particularly valuable given the fluctuating nature of psychotic symptoms and the critical need for timely interventions. Traditional clinical assessments, such as face-to-face interviews or self-reported questionnaires, which are often conducted periodically and rely on subjective reporting, may be prone to bias and fail to capture subtle or rapid changes in a patient’s psychosocial functioning (62–64). With emerging AI-driven approaches, it leveraged technologies such as mobile sensing and speech feature analysis to provide real-time, quantifiable insights (46, 48). In our review, it was found that these innovative methods offer a transformative shift from conventional practices by harnessing multi-modal data to track behavioral patterns and vocal characteristics in schizophrenia. This aligned with the growing field of digital phenotyping, where passive data from smartphones and wearables is increasingly utilized to infer mental health states, as seen in studies exploring depression and anxiety (65). However, both methods revealed limitations in generalizability—mobile sensing struggled to predict outcomes for new patients without historical data (48). At the same time, speech analysis encountered difficulties with patients whose QoL scores deviated significantly from the norm, particularly those with severe symptoms (46). This suggested that while AI can enhance monitoring, it may require personalization or larger, more diverse datasets to achieve robust performance across varied populations.

#### 5.2.3 Personalized intervention

Apart from the wide range of AI techniques and models, this review highlighted the potential of partnerships between AI methods and traditional statistical techniques. Greedy Fast Causal Inference (GFCI) and Structural Equation Model (SEM) were combined to reveal hierarchical and causal insights, identifying social affective capacity and motivation as high-impact treatment targets (51). AI models excel at processing large, high-dimensional datasets and identifying patterns, but they often lack interpretability, earning them the “black box” label. Statistical models, designed for causal inference, complement AI by providing rigorous, interpretable results that can validate cause-and-effect relationships. This combination was supported by (66), who found that integrating the two approaches yields complementary biologically meaningful conclusions.

Similarly, techniques like the Bayesian network identified a hierarchical causal chain in ToM ability, where first-order first-person ToM is the foundational ToM skill that influences performance on more complex ToM tasks (40). In the schizophrenia population group, second-order ToM and Allocentric third-person ToM are more pronounced impairments. Clinically, this implied that interventions should prioritize strengthening foundational ToM skills, such as self-reflection and awareness of one’s mental states, before progressing to more complex social cognitive tasks. Overall, the mechanistic analyzes of socio-affective capacity and motivation’s influence on functional outcomes and hierarchical ToM structures may provide clinicians with new direction for personalized treatment approaches (40, 51).

#### 5.2.4 Real-time recommendation systems

Deep reinforcement learning was employed to suggest conversation topics and therapeutic techniques by analyzing patient data during live sessions and providing therapists with tailored suggestions (52). This is especially beneficial for complex mental health conditions like psychosis, where individualized, context-sensitive care is significant (67). Surprisingly, none of the studies in this review utilized generative AI, commonly known as chatbots in the psychosocial treatment of psychosis, as Olawade et al. (68) emphasized the emerging trend of AI therapists in chatbots, for example, Woebot, Wysa, and BetterHelp in delivering psychotherapy like cognitive behavioral therapy without human involvement for anxiety and depression. However, Alliende et al. (69) raised concerns about the application of chatbots to psychosis due to inherent risks and limitations. It was found that the current common generative AI models, including chatbots like ChatGPT and Gemini, were trained on datasets that often reflect societal biases and stigmatizing views towards individuals with schizophrenia, potentially reinforcing misconceptions about violence or social distance.

Additionally, Arbanas et al. (70) found that patients with mental disorders, including psychosis, reported significantly lower satisfaction with chatbot responses compared to human therapists. Moreover, it revealed that about 50% of the participants found it more comfortable to communicate with humans than chatbots, with none perceiving chatbots as more professional or knowledgeable. This highlighted the irreplaceable role of human empathy and trust, particularly in psychosis, where therapeutic rapport is foundational. In contrast, real-time recommendation systems offered a promising alternative by equipping human therapists with AI-driven insights, preserving the human-led therapeutic process while enhancing treatment delivery. These systems could provide data-driven suggestions tailored to the patient’s needs, potentially improving outcomes without losing the human connection. However, their clinical applicability remained limited, as Lin et al. (52) reported only moderate accuracy in current systems, underscoring the need for further refinement of the AI system.

### 5.3 Implications for future AI applications & research directions

While the application of AI demonstrated potential in psychosocial functioning for psychosis, several methodological limitations have to be acknowledged to inform future research and clinical practice.

First, the generalizability of findings may be limited by specific datasets, small sample size, and consent biases. For example, five studies’ samples were particular to the Asian population, which may not represent the diversity of the global psychosis population, and vice versa. Small and varied sample sizes, ranging from 18 to 1027, affected the generalizability of findings. Additionally, consent biases may arise due to the 49% participation rate in the dataset of Leighton et al. (45), which may differ systematically from that of non-participants. Future recommendations on replicating studies in varied ethnic groups and larger, diverse cohorts may improve generalizability and reduce biased results.

Second, the quality and availability of data may affect the accuracy and reliability of AI models, particularly in terms of psychosocial function. Although the vast majority of studies utilized prospective datasets, four relied on retrospective data, which may pose a higher risk of recall and selection biases. Moreover, the only RCT included in the studies — Wang et al. (48) — was assessed as having a high risk of bias, primarily due to missing outcome data, which raises concerns about the completeness and reliability of the findings. Additionally, the predominance of cross-sectional data limited the ability to evaluate longitudinal changes, which is crucial for understanding the evolving nature of psychosocial function. To overcome these challenges, future research should prioritize prospective data collection and longitudinal analysis, which can facilitate the capture of the dynamic nature of psychosocial function in psychosis, ultimately enhancing their clinical utility and broader applicability.

Third, the design of models may impose additional constraints. Most of the models designed were static, lacking post-baseline updates or biomarkers, which limited their adaptability to individual patient trajectories. Models among studies often required inputs of specific assessment baseline scores, which may not be universally available across clinical settings. The reliance on proxy measures, such as socio-affective capacity derived from QLS items, and the untested construct validity may compromise predictive accuracy. The development of dynamic models that incorporate a broader range of variables, including additional biomarkers and standardized outcome measures, may facilitate future clinical integration.

Lastly, the risk of overfitting in ML is of concern. Given that studies predominantly employed ML as the primary approach, less than one-third externally validated their findings. The low certainty of the evidence identified using GRADE and the high risk of bias in the included RCT were primarily due to limited external validation. Also, none of the studies compared AI predictive modeling with existing clinical assessments; thus, the clinical utility of AI models remains uncertain. Future research should prioritize external validation to confirm the predictive power of these models. For future clinical integration, a comparison with traditional clinical tools is necessary to assess feasibility and effectiveness.

### 5.4 Ethical considerations

Considerations on ethics and privacy in applying AI techniques in the mental health clinical practice should be proactively managed. The included AI-augmented studies, especially the approaches of continuous sensing and real-time data processing, pose a high risk to data security and patient autonomy. However, there was a lack of studies detailing the strategies for managing patient consent or mitigating clinical errors. While anonymization was noted, the absence of specifics on data collection processes or representativeness across cultural, linguistic, or socioeconomic contexts raises ethical concerns about fairness and bias. Existing literature was consistent with these concerns: Fisher (71) discussed data security and bias in AI for psychiatry, Saeidnia et al. (72) highlighted privacy, consent, and fairness in mental health AI, and Gooding et al. (73) noted that only 15.2% of studies address ethical implications, with minimal focus on consent. Future research should address the above concerns to ensure equitable benefits without compromising patient welfare.

### 5.5 Limitations

This systematic review and meta-analysis presented certain limitations. Firstly, the relatively small number of eligible studies restricted the breadth of insights into AI applications for psychosocial functioning in psychosis, particularly given the field’s early developmental stage. This scarcity was compounded in the meta-analysis, where only partial independent studies contributed to the pooled performance metrics, thereby limiting generalizability and preventing reliable evaluation of publication bias. Secondly, substantial heterogeneity across the studies reflected variability in outcome domains, sample sizes, modeling strategies, and predictor sets such as genetic versus clinical features, limiting a comprehensive comparability of results. The overlapping cohorts involved in the meta-analyses also introduced risks of inflated precision and reduced applicability. Lastly, although the majority of included studies were considered to have good methodological rigor, two observational studies and one RCT were evaluated as of moderate quality and a high risk of bias, which may compromise the validity of the overall findings; however, sensitivity analyses confirmed that these biases did not unduly influence the results.

## 6 Conclusion

This systematic review and meta-analysis demonstrated the growing application of AI, particularly supervised ML, in addressing psychosocial functioning in psychosis. The included studies, largely of good methodological quality, demonstrated moderate overall performance in predicting outcomes, exploring underlying mechanisms, and providing therapeutic support across various domains, including social functioning, occupational functioning, social cognition, and QoL. Our findings suggested that clinical applications of AI could prioritize social cognition domains and leverage clinical symptoms for enhanced precision in early identification, personalized interventions and data-driven monitoring. Methodological advances, including the ensemble learning with feature selection, tailored models incorporating patient-specific data rather than one-size-fit-all models, and probabilistic models for handling heterogeneous data were also discovered. Overall, the remarkable potential of AI to enhance psychosocial care in psychosis was underscored. Nevertheless, the field remains in its early stages, with critical limitations on methodological consistency, data quality, model design, and ethical issues being highlighted in this review. These challenges necessitate careful consideration when applying AI in clinical practice. Given the dynamic and complex nature of psychosocial functioning in psychosis, AI should not be considered as replacing clinical expertise but rather complementing it. Human elements, including clinicians’ judgment and empathy, remain essential for delivering comprehensive psychosocial care, thereby fostering a collaborative relationship between AI technology and human insight. Future research is recommended to focus on bridging existing gaps and realizing AI’s full potential in promoting effective and ethical psychosocial care for individuals with psychosis.

## Data Availability

The original contributions presented in the study are included in the article/**Supplementary Material**. Further inquiries can be directed to the corresponding author.
