# Supplementary material for: Application of artificial intelligence and psychosocial functioning in psychosis: a systematic review and meta-analysis
Source: Front Psychiatry. 2025 Nov 5;16:1692177. doi: 10.3389/fpsyt.2025.1692177 (PMC12626789; doi:10.3389/fpsyt.2025.1692177)
Supplement: Supplementary file 1 [file Table1.docx]

**Appendix A. Supplementary Data**

| Search Strings | |
| --- | --- |
| PubMed | (("machine learning"[MeSH Terms] OR "machine learning"[Title/Abstract] OR "natural language processing"[MeSH Terms] OR "natural language processing"[Title/Abstract] OR "artificial intelligence"[MeSH Terms] OR "artificial intelligence"[Title/Abstract] OR "AI"[Title/Abstract] OR "deep learning"[Title/Abstract] OR "neural networks, computer"[MeSH Terms] OR "neural network*"[Title/Abstract] OR "chatbot*"[Title/Abstract] OR "reinforcement learning"[Title/Abstract]) AND ("psychotic disorders"[MeSH Terms] OR "psychosis"[Title/Abstract] OR "schizo*"[Title/Abstract]) AND ("social behavior"[MeSH Terms] OR "psychosocial function*"[Title/Abstract] OR "social function*"[Title/Abstract] OR "social cognition"[MeSH Terms] OR "social cognition"[Title/Abstract] OR "cognitive remediation"[Title/Abstract] OR "cognition"[MeSH Terms] OR "cognitive function"[Title/Abstract] OR "functional outcome*"[Title/Abstract] OR "quality of life"[MeSH Terms] OR "quality of life"[Title/Abstract] OR "vocational skill*"[Title/Abstract] OR "psychotherapy"[MeSH Terms] OR "psychotherapy"[Title/Abstract])) |
| Scopus | TITLE-ABS-KEY(("machine learning" OR "natural language processing" OR "artificial intelligence" OR "AI" OR "deep learning" OR "neural network*" OR "chatbot*" OR "reinforcement learning") AND ("psychosis" OR "schizo*") AND ("psychosocial function*" OR "social function*" OR "social cognition" OR "cognitive remediation" OR "cognitive function*" OR "functional outcome*" OR "quality of life" OR "vocational skill*" OR "psychotherapy")) |
| ACM Digital Library | ("machine learning" OR "natural language processing" OR "artificial intelligence" OR AI OR "deep learning" OR "neural network" OR chatbot* OR "reinforcement learning") AND (psychosis OR schizo*) AND ("psychosocial function*" OR "social function*" OR "social cognition" OR "cognitive remediation" OR "cognitive function" OR "functional outcome*" OR "quality of life" OR "vocational skill*" OR psychotherapy) |
