# Supplementary material for: Application of artificial intelligence and psychosocial functioning in psychosis: a systematic review and meta-analysis
Source: Front Psychiatry. 2025 Nov 5;16:1692177. doi: 10.3389/fpsyt.2025.1692177 (PMC12626789; doi:10.3389/fpsyt.2025.1692177)
Supplement: Supplementary file 2 [file Table2.docx]

**Appendix B. Supplementary Data**

Result of quality assessment for cohort studies:

| Criteria | Li et al. (42) | Leighton et al. (45) | de Nijs et al. (41) | Walter et al. (47) -Training cohort | Wang et al. (48) | Miley et al. (49) | Miley et al. (51) |
| --- | --- | --- | --- | --- | --- | --- | --- |
| Selection: Representativeness | ★ | ★ | ★ | ★ | ★ | ★ | ★ |
| Selection: Non-exposed cohort | NA | NA | ★ | NA | NA | NA | ★ |
| Selection: Ascertainment of exposure | ★ | ★ | ★ | ★ | ★ | ★ | ★ |
| Selection: Demonstration that the outcome of interest was not present at the start of the study | ★ | ★ | ★ | ★ | NA | ★ | ★ |
| Comparability | ★★ | ★ | ★★ | ★★ | ★ | ★ | ★★ |
| Outcome: Assessment of outcome | ★ | ★ | ★ | ★ | ★ | ★ | ★ |
| Outcome: Follow-up long enough | ★ | ★ | ★ | ★ | ★ | ★ | ★ |
| Outcome: Adequacy of follow-up | ★ | ★ | ★ | ★ | NA | NA | ★ |
| Total score (★) | 8 | 7 | 9 | 8 | 5 | 6 | 9 |
| Study quality | Good | Good | Good | Good | Fair | Good | Good |

Result of quality assessment for cross-sectional studies:

| Criteria | Badel et al. (39) | Bosco et al. (40) | Lin et al. (43) | Lin et al. (44) | Shibata et al. (46) | Lin et al. (50) |
| --- | --- | --- | --- | --- | --- | --- |
| Selection: Representativeness | ★ | ★ | ★ | NA | ★ | ★ |
| Selection: Sample size | ★ | NA | ★ | NA | NA | ★ |
| Selection: Non-respondents | NA | NA | NA | NA | NA | NA |
| Selection: Ascertainment of exposure | ★★ | ★★ | ★★ | ★★ | ★★ | ★★ |
| Comparability | ★ | ★★ | NA | NA | NA | ★ |
| Outcome: Assessment | ★ | ★ | ★★ | ★★ | ★★ | ★★ |
| Outcome: Statistical test | ★ | ★ | ★ | ★ | ★ | ★ |
| Total score (★) | 7 | 7 | 7 | 5 | 6 | 8 |
| Study quality | Good | Good | Good | Fair | Good | Good |

Result of quality assessment for RCT:

|  | Walter et al. (47) - Intervention sample |
| --- | --- |
| Domain 1: Risk of Bias Arising from the Randomization Process | Low risk |
| Domain 2: Risk of Bias Due to Deviations from the Intended Interventions (Effect of Assignment to Intervention) | Some concerns due to the lack of blinding for participants and intervention deliverers, though no clear evidence of deviations is reported. |
| Domain 3: Missing Outcome Data | High risk due to missing outcome data and without evidence of appropriate handling |
| Domain 4: Bias in Measurement of the Outcome | Some concerns due to a lack of assessor blinding |
| Domain 5: Bias in Selection of the Reported Result | Some concerns due to the absence of information on pre-specification |
| Overall Judgement | High risk of bias (primarily due to missing outcome data, lack of blinding, unclear pre-specification) |

Result of quality assessment of non-observational proof-of-concept study:

|  | Lin et al. (52) |
| --- | --- |
| Type of predication model study | Development and validation |
| Domain 1: Participants | |
| - Risk of bias | Low |
| - Applicability concern | Low |
| Domain 2: Predicators | |
| - Risk of bias | Low |
| - Applicability concern | Low |
| Domain 3: Outcome | |
| - Risk of bias | Low |
| - Applicability concern | Low |
| Domain 4: Analysis | |
| - Risk of bias | Low |
| Overall risk of bias | Low |
| Overall applicability concern | Low |
