# Supplementary material for: Application of artificial intelligence and psychosocial functioning in psychosis: a systematic review and meta-analysis
Source: Front Psychiatry. 2025 Nov 5;16:1692177. doi: 10.3389/fpsyt.2025.1692177 (PMC12626789; doi:10.3389/fpsyt.2025.1692177)
Supplement: Supplementary file 3 [file Table3.docx]

**Appendix C: Supplementary data**

GRADE Summary findings:

| **Quality assessment** | | | | | | | | | **Summary of findings** | |
| --- | --- | --- | --- | --- | --- | --- | --- | --- | --- | --- |
| Outcome | No. of Studies | Study Design | Risk of bias | Inconsistency | Indirectness | Imprecision | Publication bias | Overall quality of evidence | Total sample size | Pooled  Effect |
| AUC | 4 | RCT, Cohort, Cross-sectional | 1 High (RCT),  3 Low (cohort & cross-sectional studies) | Serious (Heterogeneity: I² = 92%; τ² = 0.0) | Serious | Not serious | Unlikely but uncertain  (Egger’s p≈0.08; only 4 studies) | ⊕⊕○○  Low —  further research very likely to impact confidence | 1939 | 0.70 (95% CI: 0.63–0.76) |
| RMSE | 2 | Cohort (only internal validation) | 1 Low,  1 Some concerns | Serious  (Heterogeneity: I² = 88.4%; τ² = 0.95) | Not serious | Serious | Not assessable  (<10 estimates) | ⊕○○○  Very low certainty — estimate of effect is very uncertain | 302  (same cohort) | 8.15 (95% CI: 7.32–8.98) |
